# Large-Scale Cultivation of Magnetotactic Bacteria and the Optimism for Sustainable and Cheap Approaches in Nanotechnology

**DOI:** 10.3390/md21020060

**Published:** 2023-01-19

**Authors:** Anderson de Souza Cabral, Mariana Verdan, Rogerio Presciliano, Felipe Silveira, Tarcisio Correa, Fernanda Abreu

**Affiliations:** Instituto de Microbiologia Paulo de Góes, Universidade Federal do Rio de Janeiro, Rio de Janeiro 21941-902, Brazil

**Keywords:** magnetosomes, bioreactor, magnetic nanoparticles, bioprocess, patent

## Abstract

Magnetotactic bacteria (MTB), a diverse group of marine and freshwater microorganisms, have attracted the scientific community’s attention since their discovery. These bacteria biomineralize ferrimagnetic nanocrystals, the magnetosomes, or biological magnetic nanoparticles (BMNs), in a single or multiple chain(s) within the cell. As a result, cells experience an optimized magnetic dipolar moment responsible for a passive alignment along the lines of the geomagnetic field. Advances in MTB cultivation and BMN isolation have contributed to the expansion of the biotechnological potential of MTB in recent decades. Several studies with mass-cultured MTB expanded the possibilities of using purified nanocrystals and whole cells in nano- and biotechnology. Freshwater MTB were primarily investigated in scaling up processes for the production of BMNs. However, marine MTB have the potential to overcome freshwater species applications due to the putative high efficiency of their BMNs in capturing molecules. Regarding the use of MTB or BMNs in different approaches, the application of BMNs in biomedicine remains the focus of most studies, but their application is not restricted to this field. In recent years, environment monitoring and recovery, engineering applications, wastewater treatment, and industrial processes have benefited from MTB-based biotechnologies. This review explores the advances in MTB large-scale cultivation and the consequent development of innovative tools or processes.

## 1. Introduction

Since the observation of magnetotactic bacteria (MTB) by electron microscopy in the 1970s [1], this group has attracted the attention of the scientific community because of their unique magnetic structure [2]. Today, MTB have become an interesting and promising biotechnological tool [3]. MTB biomineralize magnetosomes, referred to here as bacterial magnetic nanoparticles (BMNs), which are organelles composed of iron minerals, magnetite (Fe_3_O_4_), or greigite (Fe_3_S_4_) enveloped by a phospholipid bilayer membrane. These organelles remain aligned in a chain in the cytoplasm due to bacterial cytoskeleton proteins [4]. The alignment of the ferrimagnetic nanocrystals causes the magnetic moment of each crystal to sum up and confers the cell an optimized magnetic dipolar moment, a characteristic responsible for MTB’s ability to orient passively along the lines of the Earth’s magnetic field [5]. As a result, these bacteria have the ability to orient along magnetic fields lines and, with flagellar propulsion, swim through chemically stratified water columns in marine and freshwater environments [4]. This guided movement, named magnetotaxis, is used during the search for the oxic–anoxic interface (OAI), a microaerobic region in a water column or sediment where MTB’s metabolism is at a maximum [6,7,8].

The biotechnological potential of MTB has long expanded from the first application of BMNs obtained from uncultured MTB from a pond [9] primarily because of the refinement of methods of mass cultivation and BMN isolation [10,11,12]. Since then, several studies with mass-cultured MTB have allowed for an increase in the possibilities of applying not only purified BMNs but also whole cells in different biotechnological fields. Vargas and colleagues [3] reviewed original research from the last three decades and showed that the major efforts of studies using MTB and their magnetite BMNs in specific applications included cell separation, hyperthermia, drug delivery, and the contrast enhancement of magnetic resonance imaging, with other applications also being possible. Although the first papers reported the use of MTB and BMNs in biomedicine, their applicability is not restricted to this field. In recent years, environment monitoring and recovery approaches, engineering technologies, wastewater treatment, and industrial applications have benefited from MTB-based technologies. 

MTB are model systems to study biomineralization, magneto-aerotaxis, and biomimetics aiming to solve current low-yield BMN production [13,14,15]. By studying how complex biologically controlled mineralization by organisms occurs, researchers have tried to produce biomimetic magnetite nanoparticles mediated by BMN-associated proteins and increase the production of those nanoparticles in the laboratory. The mechanism of BMN synthesis relies on a unique set of genes, referred to as *mam* (magnetosome membrane) and *mms* (magnetic particle membrane-specific) genes [13,15,16]. These genes control the steps of biomineralization, such as BMN membrane formation, in the assembly of the BMN chain, in iron transport and nucleation or in the control of their size, shape, and crystal maturation [15]. Thus, different studies have described the high-yield expression of a small number of proteins codified by biomineralization-related genes through recombination using bacterial hosts with less fastidious growth and fewer purification steps [16,17,18,19,20,21]. Purified recombinant proteins retained their functionality and were used in the bioinspired synthesis of magnetite nanoparticles. Biomimetic magnetite nanoparticles are usually presented as a potential solution to increase the production of synthetic magnetic nanoparticles for technological applications. However, several problems still need to be solved when making synthetic nanoparticles, such as the lack of complete control over their size and shape, and the need for an external coating. Thus, biomimetic magnetite nanoparticles have not reproduced the qualities observed in natural BMNs [15].

BMN exhibit a narrow crystal size range, high chemical purity, and few crystallographic defects compared to synthetic iron oxide nanoparticles [3]. The stable single magnetic domain, which is a function of their nanosized dimensions, confers these nanoparticles an excellent responsiveness to magnetic stimuli. BMNs have a biocompatible lipid bilayer around each mineral particle and a high thermal and colloidal stability in addition to their ease in terms of the insertion of functional, biocompatible organic molecules [22,23,24,25]. The reproduction of the refined characteristics found in biogenic BMNs is difficult to obtain with abiotically produced magnetic nanocrystals [26,27].

Biomineralization is almost certainly determined by genetics. All the information necessary to biomineralize BMNs is on the magnetosome gene clusters, which are genomic regions on each MTB composed of genes that are transcribed together as operons [28,29]. The chemical composition and morphology of BMNs, with a few exceptions, are specific within a given species [30], and each species has control over the composition, size, shape, and direction of elongation of mineral crystals. Phylogeny is correlated to the composition of these crystals [31,32,33]. Magnetotactic Alpha- and Gamma- classes of the Proteobacteria phylum biomineralize magnetite crystals that include cuboctahedral and elongated prisms. The most studied Alphaproteobacteria species, *Magnetospirillum magneticum* strain AMB-1, *Magnetospirillum magnetotacticum* strain MS-1, and *Magnetospirillum gryphiswaldense* strain MSR-1, biomineralize cuboctahedral magnetite crystals of approximately 3–40 nm in diameter, as does the recently described *Magnetospirillum kuznetsovii* sp. nov. [32,34]. Differently, *Magnetovibrio blakemorei* MV 1^T^ biomineralizes prismatic magnetite crystals approximately 60 nm long [35]. The Deltaproteobacteria magnetotactic strain *Desulfovibrio magneticus* RS-1 biomineralize bullet-shaped magnetite crystals, and *Desulfamplus magnetovallimortis* BW-1 biomineralize bullet-shaped magnetite crystals or cuboctahedral greigite crystals or both, with magnetite crystals always being elongated-anisotropic [32,36,37,38,39]. Greigite-producing MTB form a monophyletic clade in the Deltaproteobacteria class [37]. Pósfai and colleagues [32] conducted a thorough analysis of the types of BMNs found so far, but with the in-depth study of nanoparticles in recent years, new findings have been reported. *Magnetofaba australis* strain IT-1, for example, a strain of the candidate class Etaproteobacteria, biomineralized BMNs with an elongated octahedral morphology [40]. Despite their natural occurrence in environmental water samples, only a small number of MTB have been isolated in axenic culture. MTB belonging to Nitrospirae and Omnitrophica phyla have been described using culture-independent techniques [8]. The only magnetotactic representative Omnitrophica phyla so far described, *Candidatus* Omnitrophus magneticus, is an ovoid with bullet-shaped magnetite magnetosomes organized in multiple magnetosome chains [41,42]. In the Nitrospirae phyla, nine different strains have already been described, possessing cell morphologies ranging from short vibrios and cocci to giant rods, with anisotropic bullet-shaped magnetite magnetosomes organized in chains. The most impressive representant of this group, *Candidatus* Magnetobacterium bavaricum, could have up to 1000 magnetite crystals in a single cell [8,42,43,44]. Through metagenome analysis and using genomic data from uncultured microorganisms, biomineralization genes were identified in genomic sequences belonging to Latescibacteria and Planctomycetes phyla. Nonetheless, the morphological characterization of these novel phyla and their magnetosomes is still missing [45]. In addition to phylogeny, it is known that several environmental parameters influence the morphology and composition of BMN crystals during their formation [32,46,47], and the understanding of the nature of this influence is essential for improving the cultivation of certain strains on a large scale.

Studies on magnetite biomineralization have shown that factors such as the pH, temperature, and Fe availability can affect not only MTB cells’ physiology but also the physical and microstructural characteristics of magnetite crystals. The presence of enzymes, such as periplasmic nitrate reductase (Nap), essential for the anaerobic respiration of nitrate, is also crucial for magnetite magnetosome formation under microaerobic conditions. In addition to that, the O_2_ concentration dramatically influences the biomineralization of magnetite BMN [47,48,49]. Thus far, only cuboctahedral magnetite BMNs have been used in biotechnological application studies [3,50]. The exception was reported in studies on the optimization of marine *Mv. blakemorei* strain MV-1^T^ cultivation in a bioreactor, which is interesting because its prismatic BMNs have a larger available surface area than that of the cuboctahedral BMNs of the genus *Magnetospirillum* [35,50], with this being advantageous for functionalization and biotechnological application. The only laboratory-grown greigite bacteria is *Desulfamplus magnetovallimortis* strain BW-1 [39]. In contrast to magnetite (Fe_3_O_4_)-producing species, that use water oxygen to form their crystal [51], it has been suggested that the producers of greigite crystals are related to anaerobic environments, where reducing conditions are available [52,53]. Greigite producers need to reach anoxic zones to accumulate reduced sulfur compounds that are electron donors in greigite formation [54]. This trait poses additional challenges for the cultivation of iron sulfide-mineralizing strains, as these environmental characteristics need to be reproduced in the laboratory [55].

Despite so many advances, few species of MTB have been isolated in axenic cultures [55,56], and much of the generated knowledge about the associated cell biology, nanocrystal biomineralization process, and genetics, came from species belonging to the *Magnetospirillum* genus [28,57,58]. Therefore, there is significant amount of MTB diversity yet to be explored, and the information generated through culture-independent molecular methods, aligned with the study of the physical and chemical characteristics of their natural environment, is important to the obtain pure cultures. This way, upscaling the cultivation of different MTB species up to large bioreactors could be feasible [13,23,59]. In this review, we discuss the advances in the large-scale cultivation of this diverse group and the development of biotechnological approaches based on them.

## 2. Bioreactor Cultivation Strategies

Although the isolation and maintenance of pure cultures of MTB are performed in small-scale laboratory flasks, the application of BMNs in technology will heavily depend upon large-scale upgrading. Several studies have analyzed and optimized the growth of MTB in fermenters and obtained significantly different production values according to the cultivation strategies (Table 1). Those strategies included modifications in terms of cultivation media, adjustments made to the physical–chemical culture conditions, and specialized regimes for feeding and oxygen injection. However, as evidenced in Table 1, the most common cultivation conduction for MTB in bioreactors is the fed-batch method.

Heyen and Schüler [59] have shown that low oxygen tensions (0.25 to 2 mbar) favor BMN formation. Different *Magnetospirillum* strains respond distinctly to higher oxygen tensions [59]; while strains MSR-1, AMB-1, and MS-1 grew in microaerobiosis in roughly the same intensity, with similar BMN productions, only strain MSR-1 could grow in aerobiosis. In the latter case, the cell magnetism was only half of that achieved in microaerobiosis due to hindered magnetosome synthesis under high oxygen concentrations. When batch-cultivated, MSR-1 cells were subjected to a reduction in oxygen tension from 20 to 2 mbar after reaching exponential growth. After 4 h elapsed from the oxygen shift, cell magnetism, which was undetectable up until this point, increased sharply until stationary growth. These findings have guided other works on MTB cultivation to maintain sufficiently low oxygen concentrations for cell growth while not hindering magnetite production [69]. In this sense, one strategy available in cultures is aeration with a low air flow and gradual increases in impeller rotation when oxygen concentration falls below a pre-set threshold [66]. The agitation intensifies the gas–liquid mass transfer at a pace that is governed by cell growth and, thus, oxygen consumption. This feedback-controlled aeration prevents aerobic conditions and maintains magnetite production activity. 

During the cultivation of strain MSR-1 [66], oxygen in media was depleted in exponential growth by bacterial respiration. Feeding with lactate kept the carbon source concentration high enough to sustain growth. After oxygen depletion, a feedback strategy was applied to maintain a low oxygen concentration but one that was sufficient for cell growth and BMN production.

Another strategy that has been used for MTB growth is the pH-responsive fed-batch method [62,63,64]. This strategy involves the introduction of the largest part of the carbon source in an acidic form (usually lactic acid) and requires the entire amount of the iron source to be consumed through the feeding media. Due to the acidic nature of lactic acid (pKa = 3.86) and its high concentration in solution (50–200 g/L), the feeding solution pH was within the range of 2.7–3.7. The consumption of medium nutrients by *Magnetospirillum* strains led to an increase in the culture pH. Automatically, this pH increase triggered a response to correct the pH, which was set to 6.8–7.0, through the addition of the feeding medium. Consequently, the pH was adjusted alongside the supplementation of the nutrients, including carbon and iron sources. The first report of such a strategy [64] was used to cultivate the strain MSR-1 in a 7.5-L bioreactor. The main reason for developing such a feeding regime was the observation that high concentrations of lactate inhibited cell growth [64]. Then, the supplementation of a pH control medium containing a highly concentrated carbon source during cultivation prevented cellular stress and sustained further bacterial growth. The same strategy was used in a subsequent batch carried out in a 42-L fermenter, resulting in a high cell density and the rapid growth of the MSR-1 strain, producing 83 mg/L and resulting in a 55.5 mg/L/day productivity in terms of magnetite.

The pH-responsive feeding strategy developed by Liu et al. [64] was the basis for the fed-batch cultivation published in several other works [62,63,68]. Fernández-Castané and colleagues [63] compared different concentrations of lactic acid (carbon source) and sodium nitrate (final electron acceptor) in the feed medium. The results of this optimized process suggested that the highest concentration of nitrate tested (25 g/L) in the feed led to a higher production of biomass. However, the concentration of lactic acid, although directly proportional to the magnetite production, was inversely proportional to cell growth. According the different growth experiments performed, the best results were a dry cell weight of 4.2 g/L in terms of biomass and a magnetite production of 139 mg/L of magnetite achieved at 71 h of cultivation.

Zhang and colleagues [62] obtained the highest values for production (356.5 mg/L) and productivity (178.26 mg/L/day) in terms of magnetite in MTB cultivation in a 42-L bioreactor. The feeding medium composition was altered from previous studies to substitute carbon and nitrogen sources. Sodium lactate was replaced by lactic acid and ammonium chloride by ammonia, reducing the solution osmotic potential, a factor that causes bacterial cell stress. Another novelty in that study was the semi-continuous two-stage fermentation strategy. In the first stage, cells were grown in a 7.5 L bioreactor with 5 L medium until the end of the exponential growth period for 30 h. At that point, 4.5 L of the spent medium was collected from the reactor vessel. Then, 4.5 L of fresh medium was added to the remaining 0.5 L and the second stage of cultivation was initiated. Although production and productivity were lower than when using the previous strategy, the semi-continuous process enabled the cultivation of a larger culture volume without the idle time necessary for vessel washing, seed propagation, sterilization, and inoculation.

Not only are the yields of magnetite scalable but also the characteristics of BMN are influenced by the media. For instance, Lin and Pan [70] compared the effects of agitation and oxygen presence in 48-h cultivations of strain AMB-1. Those experiments revealed a significant decrease in the average length (from 41.5 ± 15 to 33.0 ± 8.5 nm) and shape elongation (width/length ratio increasing from 0.78 ± 0.12 to 0.89 ± 0.08 nm) when cells were cultured under static anaerobiosis and aerobiosis with a 120-rpm agitation, respectively. The conditions also affected the BMN production, as cells in anaerobiosis produced 12 ± 5 BMN on average but only 7 ± 4 when grown in agitated aerobiosis. 

While most studies have assessed the influence of oxygen concentration on BMN formation in bioreactors, Olszewska-Widdrat and colleagues [71] examined the effects of the overall oxidation reduction potential (ORP). Although oxygen is a major oxidizing component, and, thus, increases the ORP, other reducing or oxidizing components are present in culture media. The results indicated that a reducing (−500 mV) medium favors magnetite formation in strain AMB-1. The average crystal diameter increased 18% from 31.5 ± 1.3 nm in neutral medium (0 mV) to 37.2 ± 0.6 nm when cells were cultivated at −500 mV. Additionally, the number of BMNs per cell length unit (i.e., 1 μm) also rose 66% from 5.48 ± 1.3 at 0 mV ORP to 9.1 ± 1.9 at −500 mV.

The magnetite purity also can be improved by culturing conditions [67]. Berny and colleagues [67] developed a minimal growth medium for the cultivation of strain MSR-1 cells. The medium composition, in which most trace elements (e.g., Mn^2+^, Co^2+^, Zn^2+^, Cu^2+^, and MoO_4_^2−^, etc.) were omitted and yeast extract was replaced by thiamine, was revised from the medium used by Zhang et al. [62]. The resulting magnetite experienced a drastic reduction in contaminating elements, such as Zn, Mn, Ba, and Al.

The possibility of inducing even slight alterations to the magnetite crystals and of improving the material purity allows for the rational planning of BMN synthesis for each different application. For pharmaceutical applications, the International Pharmacopoeia [72] established limits to metal contamination for therapeutic compounds. The magnetic properties of nanomaterials depend on their size and elongation and determine their responsiveness to external stimuli such as magnetic fields [73]. For example, in hyperthermia therapies, where magnetic nanoparticles are submitted to an alternating magnetic field (AMF), the heating rate and final temperature are functions of the size and shape of the nanoparticles [74].

Silva and collaborators [50], in one of the few works on large-scale cultivation outside the genus *Magnetospirillum*, optimized the cultivation of *Mv. blakemorei* strain MV-1^T^. This species produces magnetite crystals of prismatic morphology organized in a single chain with average dimensions of 53 ± 11 nm in terms of length and 35 ± 8 nm in terms of width [75]. In the cultivation, the strain MV-1^T^ can use several compounds as electron donors and carbon sources, either in microaerobiosis or anaerobiosis using nitrogen oxides. However, MV-1^T^ cells produce a higher number of BMNs when they grow anaerobically on N_2_O as the final electron acceptor. Under this regime, Silva and colleagues [50] reported the optimization of BMN production by strain MV-1^T^ through a regime of experimental planning. The experiments tested included multiple modifications in the liquid medium described by Bazylinski [35], which comprises ferrous sulfate (FeSO_4_) as the source of Fe^2+^. As a result, an optimal composition was determined for the cultivation medium of MV-1^T^ strain in the bioreactor. The maximum production of magnetite obtained in a 5-L volume was 22.4 mg/L in 96 h, approximately three times greater than that in the initial medium. The continuous growth strategy using MV-1^T^ increased the maximum production to 27.1 mg/L and decreased the number of non-magnetic cells [60]. The upscaling of the cultivation of *Mv. blakemorei* strain MV-1^T^ is interesting because the larger surfaces of prismatic BMNs presumably have more available binding sites for functional molecules than the cuboctahedral BMNs of *Magnetospirillum* [35], which could be advantageous for applications [50].

To sum up, different strategies are used for the large-scale cultivation of MTB, including simple-batch, fed-batch, and semi-continuous-batch strategies. The culture conditions must be well-controlled to prevent changes in the BMN characteristics, such as length, shape elongation, and purity. Cultivation strategies have been most extensively studied with respect to the *Magnetospirillum* genus, and magnetite nanoparticle productivity reached levels that encourage the establishment of an industrial plant for production. Promising results were obtained for producing prismatic magnetite using the marine *Mv. blakemorei* strain MV-1^T^. Overall, research on the large-scale cultivation of different strains of MTB is still necessary, especially for industrial-scale upgrading.

## 3. BMN Purification Methods

In biotechnological industries, the isolation of intracellular products may impose additional steps not required by extracellularly secreted molecules. In the case of BMNs, chemical and physical extraction methods have been employed as downstream steps. Chemical extraction can be achieved by incubation with NaOH and sodium dodecyl sulfate (SDS) [76]. Although chemical extraction dispenses the necessity of specialized equipment, high concentrations of NaOH may cause damages to the structure of the BMN membrane related to membrane protein denaturation and phospholipid saponification. Detergents, such as SDS and Triton, should be used carefully for this purpose because they can also partially or totally remove membrane components [77].

Ultrasonic cell crushing is one of the most diffused examples of physical methods for isolating intracellular bioproducts [78]. The lysis mechanism relies on the formation of cavities because of the incidence of high-frequency waves onto cell suspensions. These cavities release mechanical energy that physically disintegrates cell components. Ultrasonic crushing has been applied in different works for BMN isolation [3,79]. However, the scalability of this operation is difficult because of the high energy consumption [78] and occupational inconvenience involved, as the sound waves released can damage audition without proper protective equipment.

One of the biggest challenges in the production of bacterial products is to ensure the absence of lipopolysaccharide (LPS) endotoxin [80]. Endotoxins are major pyrogenic components and their presence in pharmaceutical products invalidates their commercialization and use [72]. In a scenario of the pilot or industrial-scale production of BMNs, the strict control of LPS contamination may be necessary because all known MTB are Gram-negative [81]. Despite the BMN vesicle being derived from the bacterial internal membrane, fragments of the external membrane could still bind to the BMNs during cell lysis.

**Figure 1:**
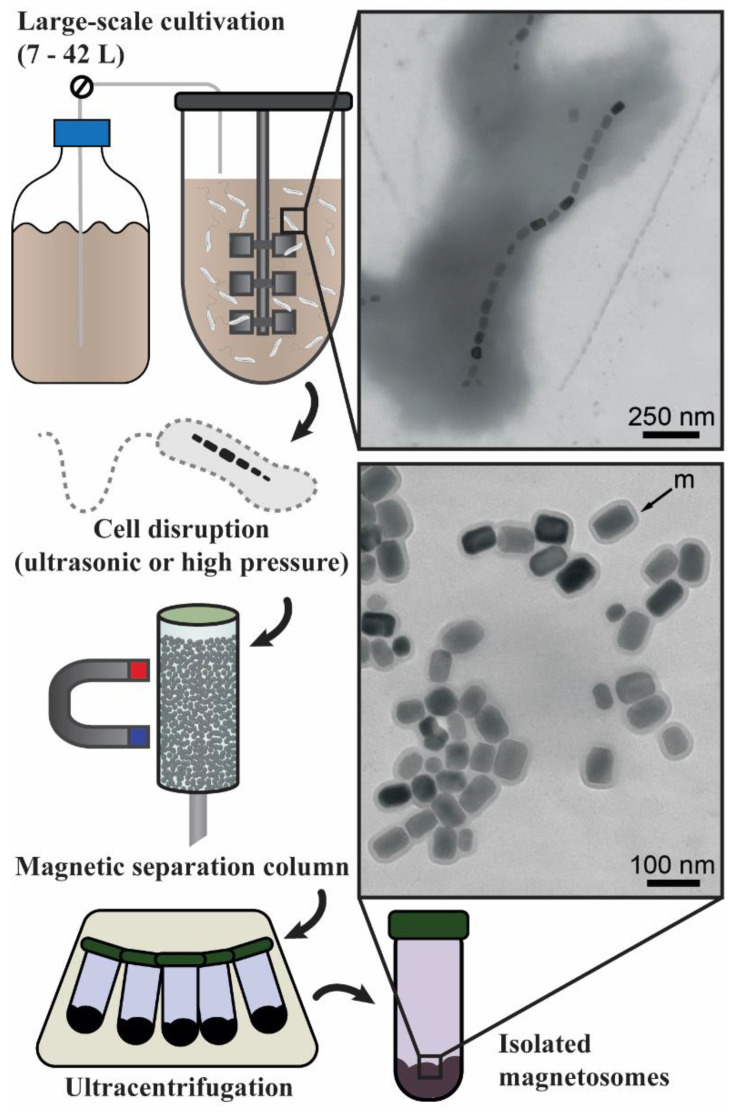
Example of a proposed process flow for the mass-production and isolation of BMNs based on the works of Guo et al. [11] and Rosenfeldt et al. [12]. Cells of MTB are cultivated in a bioreactor with volumes of liquid media ranging from 7 to 42 L. After cultivation, cells are harvested from the growth media and submitted to physical lysis through ultrasonic cell crushing or high-pressure homogenization. For the concentration of the magnetic nanoparticles, the cell lysate is percolated in a magnetizable matrix made of ferromagnetic material (i.e., steel beads), which is contained within a magnetic separation column. The magnetic concentrate is washed with an appropriate buffer (i.e., phosphate or HEPES) and is separated from the liquid phase through ultracentrifugation. The final isolated BMNs retain membrane integrity, enabling a stable colloidal dispersibility and facilitating intended applications.

For BMN purification, a physical extraction procedure using high-pressure cell lysis in a French press properly eliminates pyrogenic contamination. High-pressure lysis is commonplace in the industry and can be upscaled as a downstream section of bioreactor cultivation of MTB. Guo and colleagues [82] developed a large-scale multistep process for BMN extraction. The process comprised a high-pressure homogenizer, for cell disruption, a magnetic separation column, and a low-energy ultrasonic treatment with a urea solution, for the removal of cell debris. In this case, the magnetic separation column (MSC) was a cylinder filled with steel millimetric beads. During the elution of the magnetic cell lysate, permanent magnets were placed externally onto the column, enabling BMNs to bind to the magnetized beads. The overall technique allowed for the recovery of 300 mg of BMNs from a 6-L culture of *Ms. gryphiswaldense* strain MSR-1. The concept of an MSC was also key in the standardization of BMN isolation proposed by Rosenfeldt and colleagues [12]. This work reported a BMN extraction pipeline consisting of the high-pressure homogenization of strain MSR-1 cells derived from a 10-L culture, followed by the elution of the cell extract through an MSC and a final ultracentrifugation procedure (Figure 1). The MSC was supplied by the manufacturer and the binding medium was a ferromagnetic fiber matrix. Despite a final BMN yield of approximately 60% of the quantity within the cell extract, the contamination by cell debris was reduced to only 0.3%.

In conclusion, chemical and physical methods for BMN purification have challenges to overcome. While chemical purification diminishes the need for specialized equipment, it might damage the crystal and/or the membrane. Physical purification preserves the BMN crystal and membrane, but ultrasonic cell crushing requires higher energy consumption and extra steps to remove pyrogenic contamination. Although high-pressure approaches eliminate pyrogenic contamination, the BMN yield is compromised.

## 4. Functionalization Methods

**Figure 2:**
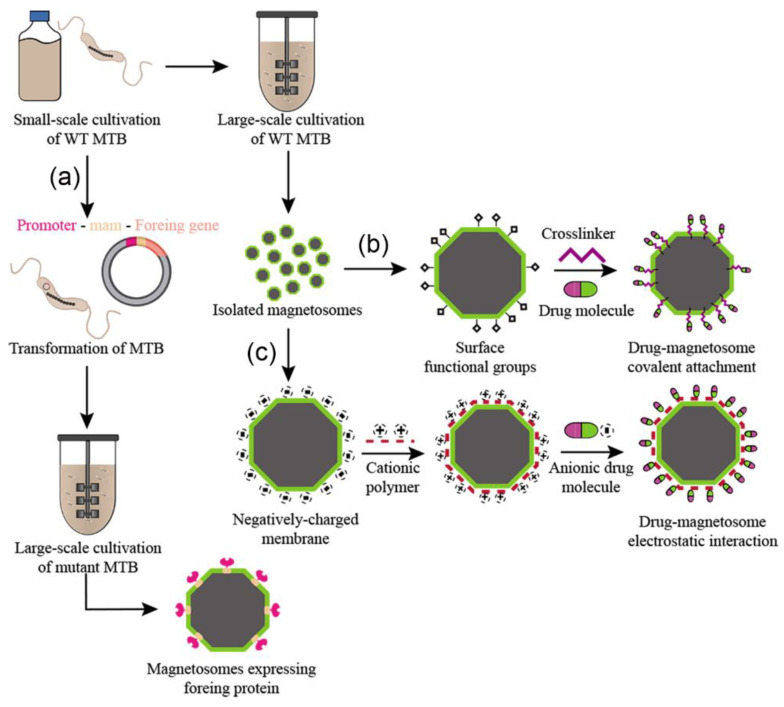
Schematic drawing shows both methods of functionalization. (**a**) Genetic functionalization; (**b**) chemical functionalization by adding an intermediate crosslinker; (**c**) chemical functionalization by adding cationic polymers.

In order to make BMNs a practical and economically viable option, we need not only to improve cultivation yields; however, in some cases, it is also necessary to develop processes to provide adequate BMNs for the desired applications. Surface modifications enable the expansion of the usability of BMNs to a wider range of biotechnological applications, and this can be achieved by either chemical or genetic methods [3]. For the rational planning of functional magnetic nanoproducts, the implications of both methods of functionalization must be considered (Figure 2). Chemical functionalization is a post cultivation process where functional molecules (i.e., drugs, proteins, genes, etc.) are attached to the BMN surface through chemical interactions [82]. Alternatively, genetic functionalization is a process in which a mutant expressing engineered proteins on the BMN surface is developed using genetic engineering techniques before mass cultivation (Figure 2a).

The phospholipids and proteins that compose BMN membranes allow for molecules such as drugs and antibodies [82,83] to be carried/loaded in the BMN structure while maintaining its innate activities. Several characteristics make BMNs a great carrier of molecules. Their high surface area leads to high loading capacities; the natural functional groups, such as the abundant amino group in their embedded proteins and negatively charged surfaces, make the attachment of foreign molecules a straightforward process [28], and their high chemical purity and crystallographic properties lead to a stable magnetic momentum and reliable magnetic response [84].

On the other hand, there is usually a lack of control over particle size and morphology in the chemical synthesis of magnetic nanoparticles. Synthetic nanoparticles usually rely on an additional coating process using polymers or lipids to improve their biocompatibility and facilitate their interaction with functional molecules [85]. In some cases, the affinity between the foreign molecule and the BMN membrane is not strong and/or stable enough for efficient loading. To work around this problem, greater carrying capacities can be achieved by adding an intermediate crosslinker (Figure 2b). A crosslinker, such as glutaraldehyde, simultaneously binds both to the BMN membrane and the target molecule, promoting either strong or weak interactions [82,86,87]. Cationic polymers, such as polyethyleneimine (PEI) and poly-L-lysine, can alter the BMN surface charge to allow for electrostatic interactions with anionic molecules (Figure 2c) [88], while crosslinkers, such as glutaraldehyde, may promote strong covalent bindings. However, the toxic effects of those molecules need to be considered for biomedical purposes [89]. For that matter, the search for biocompatible crosslinkers has become crucial [90]. Genipin is a natural molecule that is proving to be as efficient as certain common crosslinkers such as glutaraldehyde while remaining less toxic [86,91,92]. It is also worth mentioning the effect of surface modifications over the colloidal stability of BMNs, a crucial property to assert safety for in vivo applications [93].

In genetic engineering, membrane proteins that are not essential for BMN synthesis, such as *mam*A, *mam*C, *mam*F, and *mam*G, can be expressed in fusion with foreign proteins such as nanobodies, fluorophores, receptors, and enzymes [94]. In contrast to chemical methods, genetic functionalization allows for the control of the functional molecule’s binding site onto BMN proteins. Moreover, the optimization of the expression of fusion proteins increases cargo capacity and enables multiple moieties to be expressed simultaneously in the same crystal and even in the same anchor protein [95,96]. However, a major aspect to be considered is the possibility of inhibitory effects due to foreign biomolecules that may interfere in the cell metabolism and thus impair its growth [94].

Different functionalization methods might align better with certain application purposes. The use of BMNs as drug delivery platforms, evidently, depends solely on the chemical processes employed to load them with drugs [86,97] and sometimes to attach biological molecules such as small interfering RNA (siRNA), antibodies, and peptides [83,88,98]. It is worth mentioning though, that for applications such as magnetic hyperthermia, magnetic resonance imaging, and cell tracking, wild-type BMNs hold potential on their own [99,100,101], even though surface modifications can be performed in attempts to improve the capacity of BMNs for the mentioned purposes [87,98].

Apart from biomedical applications, which will be further discussed in the next section, BMNs are being genetically functionalized to display several moieties of a single or multiple enzymes on their surfaces while preserving catalytic activities and reusability [94,102]. Additionally, antibodies crosslinked to the BMN membrane can be used to detect biological markers to control food safety standards [103,104,105,106]. As aforementioned, through genetic engineering, several Mam family proteins can be used to display functional moieties. As the topmost example of this, a mutant of *Ms. gryphiswaldense* is able to synthesize BMNs with four different additional attributes: the ability to catalyze the degradation of complex carbohydrates (through glucuronidase expression) and oxidase glucose (through glucose oxidase expression), a specific binding to mCherry (through the expression of RBP nanobody), and fluorescence (through mEGFP expression) [94].

Unlike every other application mentioned here, which rely on the purification and usage of isolated BMNs, MTB cells are potential removers of metals from contaminated waters. In bioremediation, the potential of MTB comes from their unique biomineralization metabolism and the cell’s recoverability with magnetic concentration [107]. Concomitant to magnetite synthesis, it has been reported that under certain conditions, BMNs were found to be doped with transition metals (Sm, Cu, Mn, Co, and Cd) [108,109,110] and even found to synthesize individual crystals of Te and Se [107,108,109,110,111]. Like other applications, genetic engineering can bring about novel approaches as well as improve existing ones. Cell surface modifications were previously reported to increase the bioabsorption of metals [112], and a mutant able to generate cobalt ferrite nanocrystal was developed after modifications in the mms6 protein [113]. Notably, under metal-rich environments, bacterial growth can be greatly hindered, highlighting the importance of understanding the growth of the metabolism of MTB considering their application in bioremediation. Recently, it was shown that purified BMNs also can incorporate metals within their membranes [79]. The ability to re-mineralize and incorporate metals into the BMN crystal may be useful for bioremediation purposes and generate BMNs with different compositions and features. For example, hybrid silver-magnetite BMNs were obtained by incubating Ag+ with purified BMNs; the hybrid Janus-like nanoparticle presented both BMN and silver nanoparticle characteristics [79].

It is also possible to easily modify MTB cells using only the functional groups present on the membrane surface [114,115]. For example, the MO-1 strain, isolated from the Mediterranean Sea, was coated with rabbit anti-MO-1 antibodies to facilitate its attachment to *Staphylococcus aureus* cells [114]. The antibodies were specific to the MTB cell; therefore, no extra step or chemical process was necessary to prepare the cells. Similarly, the surface of *Magnetococcus marinus* strain MC-1 was decorated with drug-loaded nanoliposomes based only on direct chemical conjugation through carbodiimide chemistry [115]. In this approach, the liposomes were carboxylated, exhibiting -COOH reactive groups. They were then conjugated to MTB cells by covalently binding them to amino groups (-NH_2_) abundantly present on bacteria surface proteins [115]. Both these examples demonstrate the feasibility of chemically modifying MTB to carry molecules or structures of interest.

Nevertheless, it is crucial to highlight that most applications of whole cells do not necessarily require cell modification. For example, most bioremediation approaches using whole MTB are based on MTB biomineralization metabolism and do not require further changes in the cells [107,108,109,110]. In addition to bioremediation and biomedical applications, an experimental setup based on Faraday’s law showed the potential of both MTB cells and isolated BMNs to generate low-voltage electricity without the need for functionalization [116]. In summary, BMNs and MTB are highly versatile tools because they can be modified through chemical modifications or genetic engineering. However, they do not necessarily need modifications; purified BMNs and whole MTB cells have been directly applied solely in several studies because of their intrinsic characteristics.

## 5. Biomedical Applications

Although applicable for many uses, most published articles study the biomedical applications of BMNs [3]. Whereas there are reports of whole MTB cells in medical applications [114,115], the use and study of extracted BMNs are still preferred. This is mostly because of safety concerns over using Gram-negative bacteria in medical application due to LPS present in the bacterial cell wall, which has immunogenic properties [117,118]. Therefore, extracted and purified BMNs appear to be more suitable for biomedical purposes.

The potential of BMNs in drug delivery has already been demonstrated in a small number of studies. For example, BMNs were extracted from *Ms. gryphiswaldense* strain MSR-1 cells and used by Sun et al. [66] to produce a drug delivery system composed of BMNs with the antitumor drug doxorubicin (DOX), using glutaraldehyde as a cross-linking agent. Antitumor activity was analyzed against HL60 (human leukemia cells) and EMT-6 strains (mouse breast cancer cells). The results showed that the BMN-DOX complex displayed an intense antitumor activity once it was able to inhibit tumor cell proliferation. The complex was also stable in the circulatory system, with 80% of the DOX still attached to the BMNs after 48 h, suggesting that the drug was not fully released before reaching the target tissue, an important feature in drug delivery systems [119].

DOX was also loaded onto BMNs together with transferrin (Tf) and tested in human liver carcinoma cells (HepG2) [120]. DOX and Tf-loaded BMNs exhibited enhanced uptake by HepG2 cells in comparison with normal liver cells (HL-7702) because of the high expression of Tf receptors on the surface of HepG2 cells. The functionalized BMNs presented increased cytotoxic activity against tumor cells when compared to treatment with only DOX. The in vivo testing of DOX and Tf-loaded BMNs on mice injected with HepG2 cells showed enhanced tumor suppression rates compared to free DOX treatment. These results suggest that the nanoformulation of BMN with DOX and Tf has the ability to kill and specifically target circulating tumor cells [120].

Recently, Hafsi et al. [121] combined *Ms. magneticum* strain AMB-1 BMN and RGD peptides (BMN@RGD) to enhance X-ray and proton radiotherapy. Colorectal (DHD) and melanoma (B16F10) cancer cells, lineages known for their radioresistance, were used to investigate the BMN@RGD improvement in both modalities of radiotherapy. Cells’ sensitivity to X-rays in the presence and absence of BMN@RGD was measured. B16F10 and DHD cells presented survival rates of 81% and 75%, respectively, after a single irradiation. In the presence of BMN@RGD, cell viability reduced to 50% for B16F10 cells and 28% for DHD cells, also after one irradiation. When compared to radiotherapy with no radioenhancers, the use of BMN@RGD enhanced the effects of X-rays and protons on tumors, reducing their volume by 63% with X-ray therapy and 70% with single-dose proton therapy [121]. The results suggest that the complexes based on BMNs have great radioenhancing activity [121].

In gene therapy, BMNs were used as a delivery platform for siRNA [122]. BMNs loaded with siRNA were produced using PEI as an electrostatic binding agent, and the nanocomposites showed enhanced cellular uptake and inhibitory effects on human cervical tumor cells (HeLa) when compared to only siRNA treatment. By being internalized, the BMN-PEI-siRNA complexes were able to deliver siRNA efficiently into cancer cells and appeared to promote apoptosis since the silencing effect of siRNA was effectively expressed. Moreover, the nanocomposites gradually decreased cell viability in a dose- and time-dependent manner, supporting the potential of this type of cancer therapy [122].

The use of BMNs was also studied in local therapy for tumor treatments by magnetic hyperthermia. This therapy comprises injecting biocompatible magnetic nanoparticles into tumors and applying an AMF. The magnetic nanoparticles are induced by the AMF to increase the local temperature, which leads to the death of tumor cells as they are more sensitive to heat than healthy cells [123]. The potential of BMNs in magnetic hyperthermia therapy was evaluated in breast cancer cells using *Ms. magneticum* strain AMB-1 BMN chains [124,125] and in glioblastoma tumor using *Ms. gryphiswaldense* MSR-1 BMNs coated with poly-L-lysine [126]. In both studies, BMNs exhibited good antitumoral activity, showing great potential for their application in hyperthermia.

The potential BMNs in nanomedicine is undeniable. However, there is still an academic and regulatory gap that must be filled before practical applications. The approval of nanoparticles for medical use by regulatory agencies is a laborious and investment-demanding task. The entire approval process for nanomedicine costs approximately $1 billion per new nanomaterial, and it is a time-consuming process that may take between 10 and 15 years [127,128]. The development of a nanomedicine product must consider physicochemical characterization, biocompatibility, nanotoxicology evaluation, pharmacokinetics and pharmacodynamics assessment, process control, and scale reproducibility [129]. The characterization of a nanomaterial should be assessed throughout its development and through the different stages of its life cycle, especially if it is a nanomaterial for medical applications, in which case, the characterization should be carried in vitro and in vivo [129].

Regarding cytotoxicity, BMNs have so far presented a high level of biocompatibility when tested in vitro, as assessed in HeLa cells [76], ARPE-19 cells [130], L929 mouse fibroblasts [131,132], J774 mouse macrophage and erythrocytes [133], and H22 hepatoma cells, HL60 human leukemia cells, and EMT-6 mouse mammary cancer cells [134]. In vivo testing of BMN biocompatibility has also been performed. Sun et al. [134] injected BMNs into the sublingual veins of rats, with the median lethal dose (LD_50_) being 62.7 mg/kg. When injected 40 mg/kg, there was no observation of any major adverse effects when compared to non-treated animals. In another assay, 1 mg of BMNs injected in rabbits’ ears did not increase the animals’ body temperatures, showing that BMNs are non-pyrogenic and suggesting that they are safe in vivo [133]. The same group had previously studied the in vivo tissue distribution of BMNs in rats [134]. BMNs were injected into the sublingual veins of rats and their distribution was analyzed in the animals’ feces, urine, serum, and several organs. Of all the studied tissues and samples in the group’s work, there was the detection of BMNs only in liver cells. The livers of treated animals presented an abundance of vacuoles, which were present in the same group’s following work [134] and were suggested to be endocytosis vesicles, indicating that that is how BMNs are internalized by liver cells [135]. Tang et al. [136] labeled BMNs from *Ms. gryphiswaldense* strain MSR-1 with a fluorescent marker to investigate tissue distribution in mice. BMNs’ fluorescence signals were detected in the liver, lungs, stomach, spleen, and intestine. Transmission electron microscopy (TEM) of ultrathin sections of these organs showed that the particles were mostly found in the liver and lungs [136]. Liu et al. [137] compared mortality caused by BMNs and synthetic magnetic nanoparticles and observed that there was only one dead animal after BMN injection in the higher dose used (480 mg/kg), while synthetic nanoparticles presented a 30% and 67.70% mortality rate in the lower (135 mg/kg) and higher (240 mg/kg) doses tested, respectively. The authors also found BMNs in the spleen and liver [137]. The presence of endocytic vesicles merged with lysosomes in the liver, similar to those observed by Sun et al. [135], was also shown by the TEM of ultrathin sections of the liver.

A long-term in vivo follow up study of BMNs was carried out by Nan et al. [138] for the first time while assessing their potential as an MRI agent. An amount of ferroferric oxide, an MRI contrast agent, corresponding to 10 (8 mg/kg) and 50 times (32 mg/kg) the clinical dosage was used, and no damage was detected in the animals’ organs up to 135 days of follow-up. BMNs mostly accumulated in the liver and spleen, and a small number were found in other organs such as the heart, lung, kidney, and brain. The particles were also found in the blood, feces, and urine. Approximately half of the BMNs were discharged in feces in the first two days after administration, although the total clearance time of BMNs in mice was correlated with the amount injected into the animals, with these taking more than four months in higher dosages [138].

**Figure 3:**
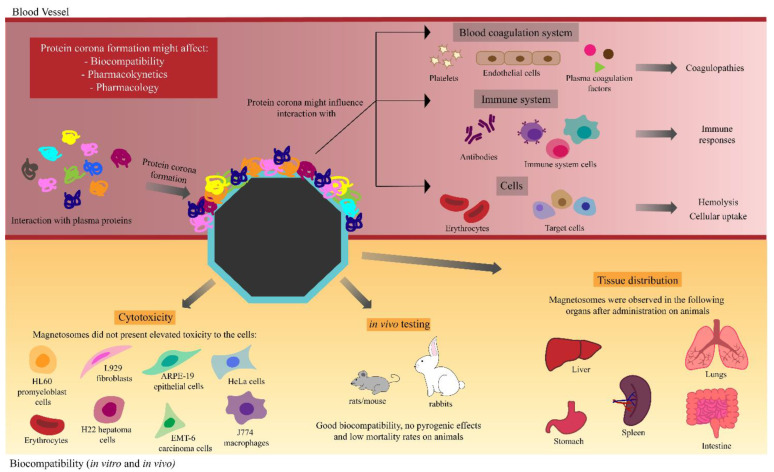
Related aspects that must be considered before the actual applicability of BMNs in nanomedicine. Protein coronas may lead to a modification in the nanoparticles’ properties such as their biocompatibility, pharmacokinetics, and pharmacology. Interactions with blood components such as cells, coagulation, and immune systems may cause specific outcomes. Regarding cytotoxicity, BMNs have so far presented a high level of biocompatibility and specific tissue distribution.

In summary, all the in vivo research so far has endorsed that BMNs are biocompatible. However, other aspects must be examined to fully evaluate biocompatibility. It is well known that when administrated to living systems, nanoparticles undergo surface modifications due to their interactions with physiological components, especially with plasma proteins [129,139,140,141]. This surface coating is called the protein corona [138] and may lead to a modification in the properties of nanoparticles such as their biocompatibility and pharmacokinetics (Figure 3) [141]. Protein corona formation may also influence nanoparticles’ cellular uptake [142] or may trigger immune responses [143] and affect hemolysis and thrombocyte activation, which is not desirable in medical approaches [144]. Therefore, it is important to study corona formation on nanomaterials used in biomedical applications.

Lai et al. [145] investigated plasma protein corona formation on BMNs obtained from *Ms. gryphiswaldense* MSR-1. It was demonstrated that the interaction between BMNs and human plasma resulted in a protein corona on the surface of the nanoparticles and that BMNs preferentially bind to a certain array of plasma proteins [145]. The corona-coated BMNs had their cellular uptake altered, with them being more internalized by endothelial cells (EC and HUVEC cell lines) compared to bare BMNs. This enhanced uptake involves the interaction between the most abundant protein absorbed onto the BMN surface, apolipoprotein E (ApoE), and surface receptors present on the membrane of the target cell, especially LDL receptors [145]. Although this was only studied ex vivo, Lai and colleagues provided valuable information for future studies on BMN interactions and behaviors in a physiological environment.

There remains a knowledge gap in our understanding of how BMNs behave in the physiological environment, especially in the context of in vivo testing, which is a key point regarding the medical application of nanoparticles. Another important factor to assess is how BMNs interact with the circulatory system and its components, particularly the blood coagulation system, as nanomaterials may cause coagulation disorders called nanoparticles-induced coagulopathies [146,147]. Nanoparticles may interact with several components of the coagulation system such as platelets, leukocytes, endothelial cells, and plasma coagulation factors, leading to coagulopathies such as thrombosis [146,147,148]. Coagulation disorders are mostly caused by multiple factors; hence, specific tests are needed to screen for the effects of BMNs on blood coagulation in vitro and in vivo [147].

An important feature for nanoparticle approval by regulatory agencies is the reproducibility and scalability of, as well as the amount of control granted in, the manufacturing process [129,146,149,150]. The manufacturing process must be well controlled since minor modifications in the process can result in alterations to the final product, compromising the safety and quality of the nanomedicine and even changing its therapeutic outcome. BMNs are produced under genetic control, which might be considered an advantage once the nanoparticle formation process is entirely controlled by MTB [151,152]. However, as described before (see item bioreactor cultivation strategies), BMN extraction has several steps such as cell lysis and BMN concentration and purification methods that must be well controlled since slight modifications might compromise the final product [129,153]. A challenging factor in the optimization of the manufacturing process is the control over these approaches at an industrial scale, as this can be inferior to at smaller scales, such as in research laboratories [129,149,153,154].

Therefore, BMNs possess great potential in biomedical applications, especially in drug delivery systems, tumor therapy, and magnetic hyperthermia. However, although they present excellent results, it is still necessary to deeply understand their interactions within a physiological environment and analyze their long-term effects on biological systems.

## 6. Technological Outputs

An important step in using BMNs for technology is ensuring that their developers can amortize the high costs necessary for its development. Thus, the amount of patent applications related to a particular technology is a strong indicator of how promising the innovation is [155]. Therefore, we performed a patent analysis to assess the generation of technology created from MTB culturing and BMNs. We searched for documents from the last 12 years using the keywords (“Magnetosome” or “Magnetosomes”) and (“Magnetotactic bacteria”) in the Derwent Innovation (Clarivate Analytics) online database. To make sure our results were within the scope of this study, we refined our findings to “Biotechnology applied microbiology” subject areas. Using this strategy, we found 182 international patents (IP), and 117 documents were considered after screening.

**Figure 4:**
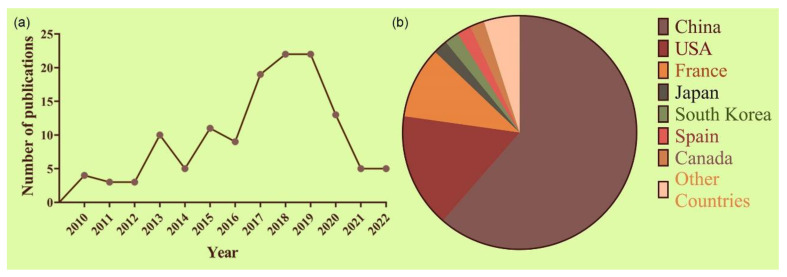
Annual patent publications of MTB and BMNs from (**a**) 2010 to 2022 and (**b**) geographical distribution of published patents from 2010 to 2022 (**b**).

The number of patent publications of MTB and BMNs in biotechnology has been growing over the last 12 years. In 2010, only 4 relevant patent applications were published (Figure 4a), while this figure was almost 6 times higher in 2018 and 2019 (with a total of 22 patents each year). It is possible to notice a drop in publications from 2020 to 2022. This effect is understandable since the social isolation measures due to the COVID-19 outbreak triggered an overall delay in non-COVID research and development [156]. It is also important to state that, at the time of writing this review, the number of patent publications for the years 2021 and 2022 may not represent the full year, since there is a 15- to 18-month time gap between submission and publication.

Figure 4b shows the geographical distribution of patent legal owners. China published the majority of patents (61.54%), indicating that this country has the largest R&D output in terms of applications of MTB and BMNs for the last 10 years. Following China, the US and France come in with 16.24% and 10.26% of the patent holders of this field, respectively. Japan, South Korea, Spain, and Canada appear next with 2.0% of patents application each.

Other criteria used in this search were the citation indices of the extracted patents, as their commercial strength, credibility, and knowledge linkage could be inferred from the number of citations [157,158]. The patent numbered WO2013106814-A1 was the most cited, with 16 citations. Next, there is a patent numbered WO2011061259-A1 with 11 citations. Patent WO2013106814-A1 concerns eukaryotic cells comprising single-celled organisms as artificial endosymbionts and methods of introduction thereof, describing how single-celled organisms carry a phenotype to eukaryotic cells that is heritable to daughter cells. The document describes eukaryotic cells containing MTB, so that MTB provides them with a magnetic phenotype that is maintained through daughter cells [159]. Patent WO2011061259-A1 claims a method for treating tumor cells and cancer by hyperthermia. The method consists of the extraction of whole BMN chains, instead of isolated BMNs, from MTB and the application of an AMF to increase antitumoral activity [124]. In addition, the invention claims that the use of certain compounds such as chelating agents and transition metals such as cobalt, nickel, copper, zinc, manganese, or chrome in the growth medium can improve the heating properties of magnetosome chains. Finally, the authors claim that the insertion of BMN chains in lipidic vesicles, which may also contain an antitumoral agent, can also improve its heating capacity and antitumoral activity [124].

**Figure 5:**
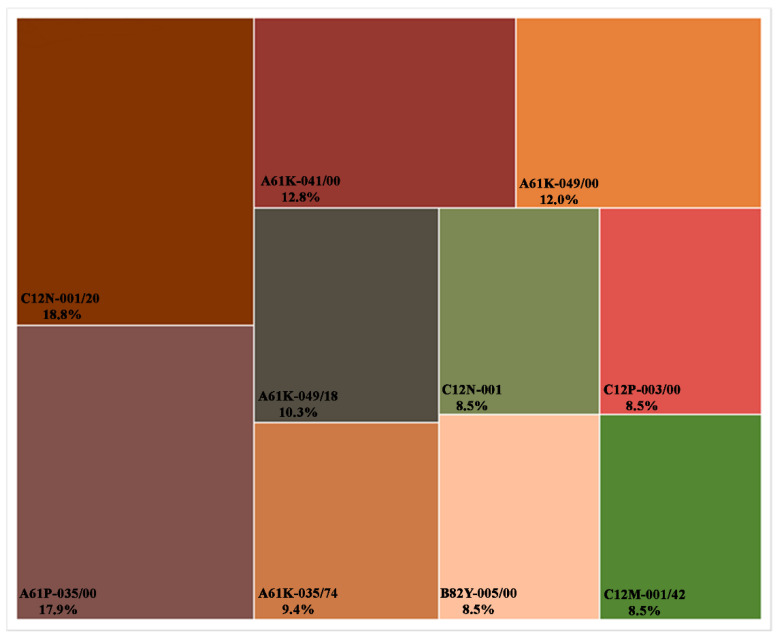
Patent distribution by technical classifications by IPC codes from 2010 to 2020.

The technical classification of filled patents was also analyzed based on the most relevant International Patent Classifications (IPCs) codes. Figure 5 shows that C12N-001/20, which represents “Bacteria; Culture Media therefor”, has the highest number of documents in this search (18.8% of all patents, 22 documents). Other relevant IPCs include “Antineoplastic Agents” (A61P-035/00, 21 patents) and “Medicinal preparations obtained by treating materials with wave energy or particle radiation” (A61K-041/00, 15 patents). The IPC codes that appeared the most and their respective descriptions can be found in Table 2.

Based on the IPC codes of the patents found, we can see that the production of MTB and BMNs is among the main nanotechnologies developed in recent years, which reflects the need for new strategies for this bioprocess. Recently, our group submitted a patent describing a method for the large-scale production of BMNs by the marine bacterium *Mv. blakemorei* strain MV-1^T^ in a bioreactor. The process is carried out initially in a fed-batch phase, followed by a chemostat culture [60,160]. With these strategies, we were able to achieve the stable production of BMNs for long periods, with high productivity, low costs, controlled physical–chemical characteristics, and avoided a decrease in magnetite production in comparison to the fed-batch strategy [60]. Additionally, the survey shows that medical applications are a trend in the development of new technologies based on BMNs. Likewise, there are published patents concerning other applications such as bioremediation [161], wastewater treatment [162], the food industry [163], and cosmetics [164]. However, these applications have a reduced number of published patents, showing that there remains a significant amount of space for the development of new technologies in these fields.

Overall, a considerable number of patents were published in the last 12 years on MTB- and BMN-based technologies. It is expected that the number of patented technologies in this field will only increase in the future, given the growing scientific research on the topic, especially due to MTB’s and BMNs’ unique properties and the significant scope in terms of fields of application.

## 7. Conclusions

Genomic information obtained with complete and partial sequences of MTB has improved our knowledge regarding appropriate substrates and contributed to the optimization of growth media, which has supported the isolation and maintenance of new strains and posteriorly mass cultivated MTB [23,165]. Regarding studies on the optimization of the growth of MTB, strategies ranged from changes in the culture media to adjustments in the physical–chemical conditions of cultures and specialized regimes of feeding and gas injection. Our review gathered information that supports the notion that modifying cultivation conditions not only improves the of BMN yield but also influences the characteristics of these nanoparticles. One such moldable characteristic is the purity of BMN crystals, which can aid in the rational and specific planning of the synthesis of BMNs and their consequent use. Another important feature of magnetosomes is their ability to be naturally coated with a biological membrane whose phospholipids and proteins allow for different molecules to be bound to the crystal while maintaining its innate activities [4]. Several experiments showed that MTB cultivation and subsequent BMN extraction were successful, although these procedures are still restricted to the cultivation of strains of the genus *Magnetospirillum*. The only exception to the use of *Magnetospirillum* species in large-scale cultivation approaches was the optimization of the large-scale cultivation of *Mv. blakemorei* strain MV-1^T^ [50,158,159]. Indeed, the low-cost production achieved for *Magnetospirillum*, which was 16,86 USD per gram of magnetosomes [62], is still outstanding and difficult to beat using strain MV-1^T^. However, the wider surface area of the prismatic magnetosomes produced by *Mv. blakemorei* strain MV-1^T^ might provide advantages in specific approaches. In addition, other cultured marine MTB species could offer BMNs with different shapes and surface properties, such as marine cocci and spirilla.

In parallel with cultivation improvements, processes for adapting MTB and BMNs to different applications have been studied. The use of BMNs in nanotechnology is favored due to their magnetic properties as well as their functional and versatile natural biocompatible coatings. Most published articles describe the use of BMNs in biomedical applications, especially concerning cancer treatment. In this regard, studies have shown that BMNs exhibited good antitumor activity, both through magnetic hyperthermia and the delivery of antiproliferative drugs. However, there is still a regulatory gap that must be filled, considering physicochemical characterization and nanotoxicology evaluations.

Despite these difficulties, the increase in studies and registrations of patents in recent years indicates the growing interest in using both MTB and BMNs in different biotechnological areas. Each step on this path is essential. The knowledge acquired so far has shown that using BMNs as technology tools passes through the specific expertise of obtaining and cultivating MTB.

## Figures and Tables

**Table 1:** Summary of large-scale cultivation of MTB in bioreactor described in the literature and their respective production and productivity values. Production and productivity values reference the magnetite mass. SB = simple-batch; FB = fed-batch; SC = semi-continuous; C= continuous.

Species/Strain	Process Conduction	Production (mg/L)	Productivity (mg/L/day)	Fe Source	Fe Concentration (µM)	Fe Feeding	Reference
*Mv. blakemorei*/MV-1^T^	FB	26	3.2	Ferrous sulfate	100	Yes	[50]
*Mv. blakemorei*/MV-1^T^	SB	22.4	5.6	Ferrous sulfate	100	No	[50]
*Mv. blakemorei*/MV-1^T^	FB	24.5	16.8	Ferrous sulfate	100	Yes	[60]
*Mv. blakemorei*/MV-1^T^	C	27.1	22.7	Ferrous sulfate	100	Yes	[60]
*Ms. magneticum*/AMB-1	FB	9	3.7	Ferrous sulfate	33	Yes	[61]
*Ms. gryphiswaldense*/MSR-1	FB	225.53	112.77	Ferric chloride	71.3	Yes	[62]
*Ms. gryphiswaldense*/MSR-1	SC	168.3	83.5	Ferric chloride	101.2	Yes	[62]
*Ms. gryphiswaldense*/MSR-1	FB	139	47	Ferric citrate	100	Yes	[63]
*Ms. gryphiswaldense*/MSR-1	FB	83.23	55.49	Ferric citrate	100	Yes	[64]
*Ms. gryphiswaldense*/MSR-1	FB	58.4	--	Ferric citrate	60	Yes	[65]
*Ms. gryphiswaldense*/MSR-1	FB	41.7	16.7	Ferric citrate	60	Yes	[66]
*Ms. gryphiswaldense*/MSR-1	FB	8–10	3.8–4.8	Ferric chloride	--	Yes	[67]
*Ms. gryphiswaldense*/MSR-1	SB	7.9	6.3	Ferric citrate	100–150	No	[59]
*Ms.* sp./ME-1	FB	120	58.7	Ferric citrate	500	Yes	[68]

**Table 2:** Different technical classifications by IPC codes of patents published from 2010 to 2020.

IPC Codes	Patents	Description
C12N-001/20	22	Bacteria, Culture media therefor
A61P-035/00	21	Antineoplastic agents
A61K-041/00	15	Medicinal preparations obtained by treating materials with wave energy or particle radiation
A61K-049/00	14	Preparations for testing in vitro
A61K-049/18	12	Preparations for testing in vitro, characterized by a special physical form, e.g., emulsions, microcapsules, liposomes
A61K-035/74	11	Medicinal preparations containing materials or reaction products thereof with undetermined constitution, Bacteria
C12N-001/21	10	Microorganisms, e.g., protozoa; modified by introduction of foreign genetic material
C12P-003/00	10	Preparations of elements or inorganic compounds except carbon dioxide
B82Y-005/00	10	Nanobiotechnology- or Nanomedicine, e.g., protein engineering or drug delivery
C12M-001/42	10	Apparatus for the treatment of microorganisms or enzymes with electrical or wave energy, e.g., magnetism, sonic wave

## Data Availability

The authors state that no new data were created in this review.
